# Supplementary material for: Internode Length Is Correlated with GA3 Content and Is Crucial to the Harvesting Performance of Tea-Picking Machines
Source: Plants (Basel). 2023 Jun 30;12(13):2508. doi: 10.3390/plants12132508 (PMC10347219; doi:10.3390/plants12132508)
Supplement: Supplementary file 1 [file plants-12-02508-s001.zip › plants-2448593-supplementary.pdf]

Table S1 Primers used in this study

Gene name	Forward primer sequence (5’-3’)	Reverse primer sequence (5’-3’)
CsGA20ox (TEA011661)	CTGGGGATTATGGAGCTTTT	TTAAAGAGGTTGGATCGCAA
CsGA3ox1 (TEA012353)	GATTTCAAAGGGGCTAATGC	GACCTCTAAACCACTTGTGT
CsGA3ox2 (TEA001361)	AAATAGCCTTCTCAACAGCA	TCGAGTGGTGATCCAAAAAT
CsGADPH	TTGGCATCGTTGAGGGTCT	CAGTGGGAACACGGAAAGC
